# Outcomes With First-Line PD-1/PD-L1 Inhibitor Monotherapy for Metastatic Renal Cell Carcinoma (mRCC): A Multi-Institutional Cohort

**DOI:** 10.3389/fonc.2020.581189

**Published:** 2020-10-22

**Authors:** Pedro Barata, Whitley Hatton, Arpita Desai, Vadim Koshkin, Ellen Jaeger, Charlotte Manogue, Patrick Cotogno, Malcolm Light, Brian Lewis, Jodi Layton, Oliver Sartor, Arnab Basu, Deepak Kilari, Hamid Emamekhoo, Mehmet A. Bilen

**Affiliations:** ^1^Deming Department of Medicine, Tulane University Medical School, New Orleans, LA, United States; ^2^Department of Medicine, University of California, San Francisco, San Francisco, CA, United States; ^3^Department of Internal Medicine, University of Alabama at Birmingham, Birmingham, AL, United States; ^4^Department of Medicine, Medical College of Wisconsin Cancer Center, Milwaukee, WI, United States; ^5^Department of Medicine, University of Wisconsin Carbone Cancer Center, Madison, WI, United States; ^6^Department of Hematology and Medical Oncology, Emory University School of Medicine, Atlanta, GA, United States

**Keywords:** metastatic renal cell carcinoma, immunotherapy, PD-1/PD-L1 inhibitor, monotherapy, first-line treatment

## Abstract

**Introduction:** The treatment landscape of metastatic renal cell carcinoma has advanced significantly with the approval of combination regimens containing an immune checkpoint inhibitor (ICI) for patients with treatment-naïve disease. Little information is available regarding the activity of single-agent ICIs for patients with previously untreated mRCC not enrolled in clinical trials.

**Methods:** This retrospective, multicenter cohort included consecutive treatment-naïve mRCC patients from six institutions in the United States who received ≥1 dose of an ICI outside a clinical trial, between June 2017 and October 2019. Descriptive statistics were used to analyze outcomes including objective best response rate (ORR), progression-free survival (PFS), and tolerability.

**Results:** The final analysis included 27 patients, 70% men, median age 64 years (range 42–92), 67% Caucasian, and 33% with ECOG 2 or 3 at baseline. Most patients had intermediate risk (85%, IMDC) with clear cell (56%), papillary (26%), unclassified (11%), chromophobe (4%), and translocation (4%) RCC. All patients had evidence of metastatic disease involving the lungs (59%), lymph node (41%), CNS (19%), liver (11%), adrenal gland (11%), and bone (11%). The median time on ICI was 3.1 (0.1–26.8) months, and the median PFS was 6.3 (95% CI, 0–18.6) months. Among the 21 patients with an evaluable response, the best ORR was 33%, including two complete responses and five partial responses. The ORR was 29% (*n* = 1 complete response, *n* = 5 partial response) in clear cell and 5% (*n* = 1 complete response) in non-clear cell RCC. Adverse events (AEs) of any cause were reported in 37% and included fatigue (11%), dermatitis (11%), diarrhea (7%), and shortness of breath (7%). Significant AEs (30%) included shortness of breath (7%), acute kidney injury (4%), dermatitis (4%), *Clostridium difficile* infection (4%), cerebrovascular accident (4%), and fatigue (7%). Three patients discontinued therapy due to grade 4 AEs.

**Conclusions:** In this multi-institutional case series, single-agent ICI demonstrated objective responses and was well tolerated in a heterogeneous treatment-naïve mRCC cohort. ICI monotherapy is not the standard of care for patients with mRCC, and further investigation is necessary to explore predictive biomarkers for optimal treatment selection in this setting.

## Introduction

Kidney cancer incidence has been increasing, and in 2020, it is estimated that there will be 73,750 new cases in 2020 in the United States (1). Renal cell carcinoma (RCC) is the most common form of kidney cancer, and the overall prognosis is particularly poor for patients who present with metastatic RCC (mRCC) (1, 2).

The treatment of mRCC has advanced significantly with the emergence of targeted agents and immunotherapies. After the approval of the programmed cell death 1 (PD-1) checkpoint inhibitor nivolumab for previously treated mRCC patients based on the phase 3 CheckMate025 study (3), combination regimens that include an immune checkpoint inhibitor (ICI) targeting PD-1, programmed death-ligand 1 (PD-L1), and cytotoxic T-lymphocyte antigen 4 (CTLA-4) have been investigated in treatment-naïve mRCC, in large phase 3 trials. The combination of nivolumab and ipilimumab has an established role in the treatment of intermediate- and poor-risk patients (CheckMate 214), and different combinations of ICIs with angiogenic therapies such as axitinib with pembrolizumab (KEYNOTE-426) and axitinib with avelumab (Javelin 101), among others, have shown significant activity in patients with mRCC (4–7). While these combinations offer a significant clinical improvement vs. sunitinib in the frontline space, adverse events (AEs) are observed in most cases and treatment interruptions, dose reductions, and permanent discontinuation are not uncommon.

The activity of single-agent ICIs in the frontline setting is under investigation. In a single-arm phase 2 study (KEYNOTE-427), the PD-1 inhibitor pembrolizumab has shown encouraging antitumor activity in patients with treatment-naïve mRCC with both clear cell and non-clear cell histology (8, 9). Two different phase 2 studies (HCRN GU16-260; OMNIVORE) testing nivolumab followed by salvage ipilimumab in patients with clear cell mRCC have also shown clinical activity of nivolumab monotherapy in this setting (10, 11).

Yet, little information is available regarding the activity of single-agent ICIs for patients with previously untreated mRCC outside of a clinical trial. We aimed to describe the utilization and outcomes with monotherapy ICI for first-line treatment of mRCC patients.

## Methods

Patients diagnosed with treatment-naïve mRCC from six academic centers in the United States (Emory University, Tulane University, University of Alabama, Medical College of Wisconsin, University of Wisconsin and University of California San Francisco) and treated with a frontline ICI between June 2017 and October 2019 were included in this retrospective analysis. Patient data were collected in compliance with the IRB guidelines of each participating institution. All consecutive patients with advanced RCC that met eligibility criteria were included in this study, based on the institutional databases at all participating sites.

Patient eligibility criteria for this analysis included pathologic confirmation of RCC, evidence of unresectable or metastatic disease using conventional CT scans, treatment with at least one dose of any PD-1 or PD-L1 drug (nivolumab, pembrolizumab, atezolizumab, and avelumab), and available baseline patient and disease information. Patients previously treated with an anti-angiogenic drug for more than 2 weeks were excluded as well as prior treatment with any PD-1/PD-L1 targeting drug, anti-CTLA-4, IL-2, or interferon-alpha. Patients treated with frontline PD-1 or PD-L1 drug as part of a clinical trial were excluded from final analysis.

Patients were followed from treatment start until death or last follow-up. Demographic, clinical, and treatment data for each patient were obtained from retrospective chart review by investigators at each institution. Patients' comorbidities considered clinically significant by the investigator at each institution were collected.

Objective best response rate (ORR) was defined by the investigator at each investigator site based on available radiographic information per RECIST. Progressive disease included radiographic and clinical progression. Clinical progression was defined by deterioration of performance status leading to best supportive care/hospice or death in patients without restaging scans available at time of analysis. Duration of response was defined for patients achieving complete or partial response from the initial documentation of response until documentation of progressive disease or until patient was lost to follow-up. Progression-free survival (PFS) was defined as the time from initiation of treatment to the time of progression or death, while overall survival (OS) was defined as the time from initiation of treatment until time of death or date of last follow up. Data on AEs were collected from clinical annotations during routine visits. Reported PD-L1 status (qualitative) data were based on the tumor tissue next-generation sequencing CLIA-certified assay available at each center; a PD-L1 status was considered positive if at least one assay detected PD-L1 expression. Significant AEs included grade 3 or above per treating investigator's assessment. The Kaplan–Meier method was used for survival analyses and two-sided *p* < 0.05 was considered significant. Statistical analysis was performed using SPSS Statistics version 23 (IBM Corp).

## Results

### Baseline Patient and Disease Characteristics

A total of 34 patients with mRCC were initially identified. Seven patients received immunotherapy as part of a clinical trial and were excluded from this dataset. The final analysis included 27 patients with median age 64 (range 42–92), 70% male, 67% Caucasian, and 33% with ECOG 2 or 3. Most patients (81%) were reported to have some type of coexisting comorbidities, which most commonly included hypertension (*n* = 7, 26%), obesity (*n* = 4, 15%), and diabetes (*n* = 5, 19%). Germline testing results were available in only four patients and detected a pathogenic alteration in SMARCB1 in one patient with unclassified RCC and negative family history of cancer, and a variant of unknown significance in one patient with clear cell RCC (ccRCC).

The study cohort consisted of patients with clear cell (*n* = 15), papillary (*n* = 7 including 3 patients with clear cell component), unclassified (*n* = 3), chromophobe (*n* = 1), and translocation (*n* = 1). One patient with papillary tumor had incipient sarcomatoid features. Four patients (4/10) had PD-L1+ tumors. Most patients (85%) had intermediate risk disease (IMDC prognostic score) and 19 underwent nephrectomy prior to initiation of any systemic treatment, including five patients who underwent cytoreductive nephrectomy. One patient with ccRCC underwent a nephrectomy after approximately 7 months after starting on frontline nivolumab with stable tumor regression. All patients had evidence of metastatic disease, most commonly involving the lungs (59%), lymph node (41%), brain (19%), liver (11%), adrenal gland (11%), and bone (11%). The most common reasons for considering a PD-1/PD-L1 monotherapy were physician's choice (41%), ECOG performance status (30%), and patients' comorbidities (15%). In four cases (15%), a combination regimen was offered, but the patient opted to start on single-agent PD-1 inhibitor due to safety concerns. Table 1 summarizes the baseline patient and disease characteristics.

**Table 1:** Baseline characteristics of the study cohort (*n* = 27).

		***n* (*%*)**
Median age (range)		64 (42–92)
Gender	Male	19 (70)
	Female	8 (30)
ECOG PS	0	6 (22)
	1	9 (33)
	2/3	9 (33)
	Unknown	3 (11)
Race	Caucasian	18 (67)
	African American	9 (33)
Histology	Clear cell	15 (56)
	Papillary	7 (26)
	Unclassified	3 (11)
	Chromophobe	1 (4)
	Translocation	1 (4)
IMDC risk	Intermediate	23 (85)
	Poor	4 (15)
IMDC risk factors		
1		9 (33)
2		14 (15)
3		4 (15)
Nephrectomy	Yes	19 (70)
	No	8 (30)
Location of metastases (>5%)	Lung	16 (59)
	Lymph node	11 (41)
	Brain	5 (19)
	Adrenal gland	3 (11)
	Liver	3 (11)
	Peritoneum	3 (11)
	Bone	2 (7)

*LLN, lower limit of normal; ULN, upper limit of normal*.

### Frontline ICI Response Rates and Outcomes

Patients received an ICI (*n* = 20 nivolumab, *n* = 6 pembrolizumab, and *n* = 1 avelumab) for their first line of systematic treatment for the initial diagnosis of stage IV disease. Median time from diagnosis of metastatic disease to starting ICI was 2.0 (0.1–24.4) months. One patient with ccRCC was previously treated with pazopanib for a total duration of 1 week prior to initiation of immunotherapy and discontinued due to AEs.

**Figure 1:**
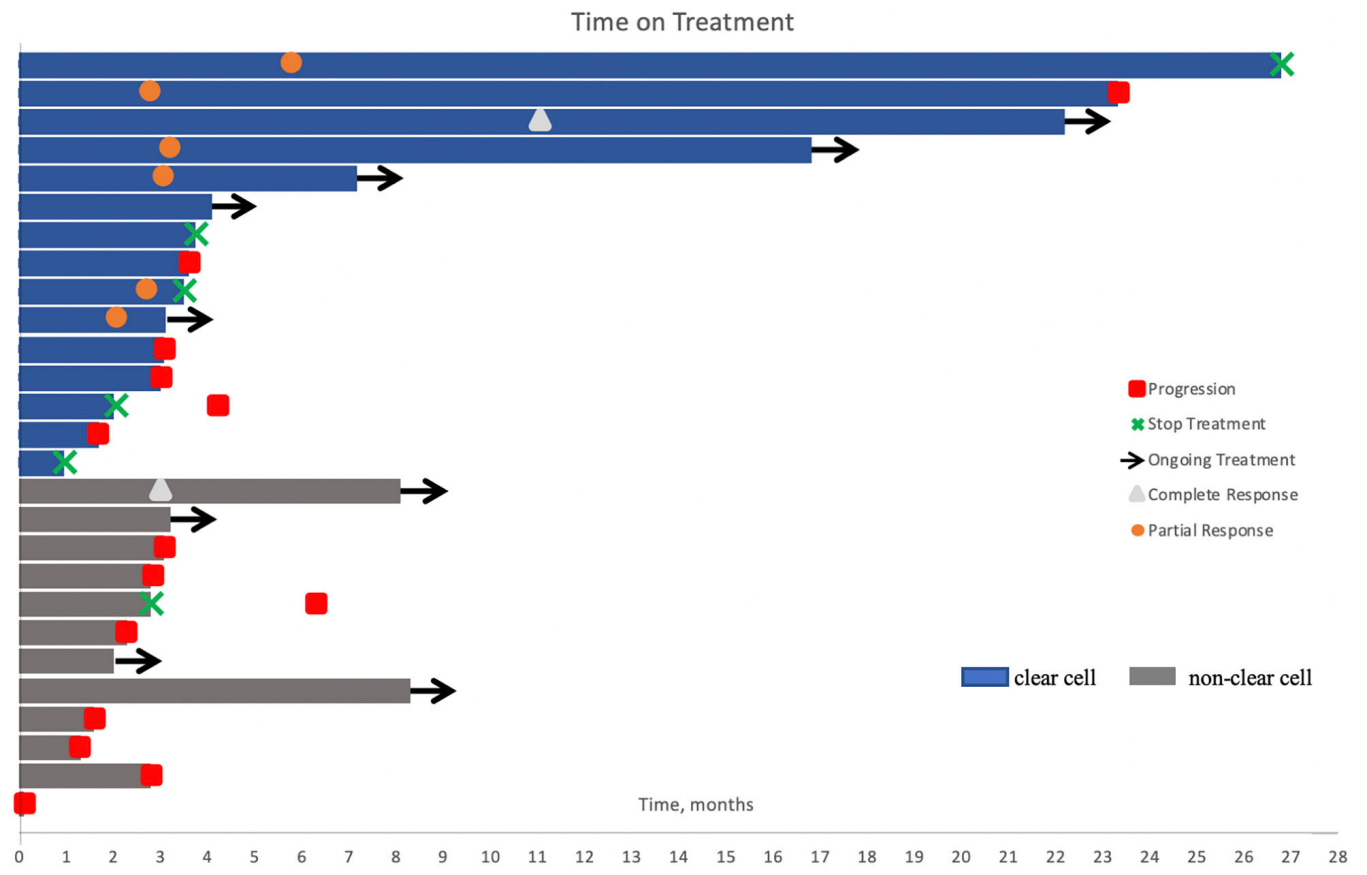
Swimmer's plot of time on treatment for the study cohort (*n* = 27). One patient received a single dose of immunotherapy. Nine patients were still on treatment at the time of analysis. Six patients had stable disease, including one patient with papillary RCC where axitinib was added to his treatment regimen after three cycles of pembrolizumab. Treatment was held or permanently discontinued in five patients due to adverse events and in one patient after 2 years of treatment.

With a median follow up of 10.7 months, the median time on immunotherapy was 3.1 (0.1–26.8) months (Figure 1).

At time of cutoff analysis, nine patients were still on treatment; axitinib was added to pembrolizumab in one patient who was still responding to therapy. Treatment interruption and discontinuation due to AEs occurred in five patients (Figure 1). Among the 21 patients with an evaluable response, the best ORR was 33%, including two complete responses and five partial responses, mostly in patients with ccRCC. The median time to best response was 3.1 (2.1–11.0) months. No responses were observed in patients with unclassified and translocation histology. Table 2 summarizes the best response rate by histology. Among the five patients who were not evaluable for response (*n* = 3 clear cell, *n* = 1 chromophobe, and *n* = 1 unclassified), all had evidence of clinical progression as best treatment response. Additionally, we excluded from the efficacy analysis one patient with prior exposure to pazopanib who achieved partial response as best response. The ORR was 29% (complete response, *n* = 1; partial response, *n* = 5) in clear cell and 5% (complete response, *n* = 1) in non-clear cell RCC (nccRCC). There were six patients with stable disease, including one patient that achieved stable disease on pembrolizumab before adding axitinib.

**Table 2:** Best response rate by mRCC histology in patients with evaluable response (*n* = *21*).

	**Clear cell**	**Non-Clear cell**			**Total**
	**(*n* = 11)**	**(*****n*** **=** **10)**			**(*n* = 21)**
		**Papillary**	**Unclassified**	**Translocation**	
		(*n* = 7^*^)	**(*n* = 2)**	**(*n* = 1)**	
ORR	6 (29%)	1 (5%)	-	-	7 (33%)
CR	1 (5%)	1 (5%)	-	-	2 (10%)
PR	5 (24%)	-	-	-	5 (24%)
SD	2 (10%)	3 (14%)	1 (5%)	-	6 (29%)
PD	3 (14%)	3 (14%)	1 (5%)	1 (5%)	8 (38%)

* *This group includes three patients with clear cell component*.

In the group of patients with CNS metastasis (*n* = 5) and evaluable response (*n* = 4), ORR was 25% (1/4 patients). One partial response was observed in ccRCC, while two patients with nccRCC had stable disease and one patient (ccRCC) had progressive disease as best response.

A total of 14 patients had disease progression and the median PFS was 6.3 (95% CI, 0–18.6) months. Patients with a complete or partial response to therapy had a significantly longer PFS compared with those who achieved stable disease or progressive disease (*p* = 0.030). Patients with ccRCC had a significantly longer PFS compared with non-clear cell histology (*p* = 0.038).

Four patients with ccRCC have been without progressive disease for more than a year (13.9–36.6 months), including two patients with complete response and two patients with partial response as their best response. On the other hand, most patients who progressed within 3 months of treatment start had non-clear cell histology (*n* = 6/8).

Among all patients who progressed on therapy (*n* = 14), subsequent treatment included a TKI [cabozantinib (*n* = 4), pazopanib (*n* = 3), and sunitinib (*n* = 1)], while six patients transitioned to hospice care or passed away. The estimated median OS was 31 (95% CI, 0–64.1) months with most patients (74%) alive at time of data cutoff.

### Safety and Treatment-Related AEs

AEs of any cause were reported in 10 patients (37%). The most common AEs included fatigue (*n* = 3), dermatitis (*n* = 3), diarrhea (*n* = 2), and shortness of breath (*n* = 2). Significant AEs (*n* = 8) included shortness of breath (*n* = 1 grade 4; *n* = 1 grade 3), acute kidney injury (*n* = 1 grade 4), dermatitis (*n* = 1 grade 4), intestinal infection due to *Clostridium difficile* (*n* = 1 grade 4, which was not considered immune-mediated), cerebrovascular accident (*n* = 1 grade 4), and fatigue (*n* = 2 grade 3).

Two patients had treatment interruption and three patients discontinued therapy due to grade 4 events (dermatitis, intestinal infection due to *C. difficile*, and cerebrovascular accident) (Figure 1).

## Discussion

In this multicenter cohort study, we found that ICI monotherapy was associated with an ORR of 36% in patients with previously untreated mRCC, driven by responses in clear cell tumors. The observed response rate and the mPFS were very similar to the reported efficacy in the prospective, first-line phase II studies KEYNOTE-427 (pembrolizumab) and the HCRN GU16-260 (nivolumab), particularly in the intermediate/poor-risk disease groups (7, 9).

Compared with ccRCC, ICIs might be less active in non-clear cell tumors (9, 12). We observed one complete response in a non-clear cell tumor. The low response rate might be the result of several factors such as the small number of patients with evaluable responses and the low rate of sarcomatoid features, which is associated with higher responses to ICI (13). Additionally, the characteristics of the patient population in this dataset included a poor performance status and brain metastases at presentation compared with other studies, which may help explain the lower clinical efficacy (14, 15). Additionally, no favorable risk patients were included and the median number of adverse risk factors of the patient population was 2, which is associated with worse clinical outcomes (16). Yet, one complete response was observed in a papillary RCC patient that is ongoing at 7 months on therapy, in line with other studies reporting objective responses that tend to last several months (8, 9).

Combination regimens containing ICIs became the standard of care for patients with mRCC based on phase 3 clinical trials conducted mainly in clear cell tumors (17). The same or similar combinations are being investigated in non-clear cell histologies, and emerging data are promising (18). Yet, not all patients will be considered eligible for those regimens. In this real-world dataset, patients' clinical functional status measured by ECOG, number of comorbidities, and concerns with safety impacted the decision to offer an ICI instead of a combination regimen or a TKI monotherapy. Of note, among six academic institutions, the small number of patients offered IO monotherapy reflects the rarity of this strategy.

More patients were treated with nivolumab compared with pembrolizumab and avelumab given the earlier FDA approval of nivolumab/ipilimumab (April 2018) followed by the pembrolizumab/axitinib (April 2019) and avelumab/axitinib regimen (May 2019).

In this cohort, the proportion of significant AEs seemed to be higher compared with prior safety data reported in the clinical studies with single-agent ICIs, which may be explained by the differences of the patient population included. ICI-related attribution was not reported for the AEs observed in our study, but patients on immune therapies who often develop autoimmune dermatitis and colitis with superimposed *C. difficile* infection have also been observed in such cases (19).

The differential activity of ICIs in this setting supports further investigation on putative predictive biomarkers to help guide treatment selection. While no RNA data were available on this dataset, gene expression signatures from mRCC in the Javelin 101 and IMmotion151 trials have demonstrated an association between the angiogenic or immunogenic signature and responses to targeted and/or immunotherapies, respectively (6, 7, 20, 21). Similarly, T-cell gene expression profile was associated with responses to pembrolizumab in KEYNOTE-427 study (22). This information has the potential to help define the role of upfront TKI or ICI monotherapy vs. combination regimen with ICI/ICI or ICI/TKI.

A different treatment strategy based on individual response to upfront ICI monotherapy is being explored (HCRN GU16-260, OMNIVORE) by allowing treatment interruption to responders while adding other ICI as salvage treatment to non-responders (10, 11). Preliminary results in patients with treatment-naïve clear cell mRCC demonstrated some activity of ICI monotherapy with an observed ORR of 29% in the HCRN GU16-260 trial and 12% at 6 months in the OMNIVORE study, respectively. Additionally, the addition of a second agent salvaged a subset of the non-responders.

Finally, the optimal sequence of systemic therapies for patients who progressed on frontline ICIs is still under investigation. In this cohort, cabozantinib was the TKI of choice after progression, which is supported by published data demonstrating the activity of this and other TKIs, such as axitinib, in the post-PD-1/PD-L1 setting (23–25).

The limitations of this case series include the retrospective nature of the analysis and the small sample size including a heterogenous group of patients which limits definitive conclusions. The data on attribution of AEs was largely not available. Nonetheless, these results provide support for ICI monotherapy in patients who are not good candidates for upfront combination regimens in a real-world setting, while we await more data from prospective studies to become available in the near future.

## Conclusions

In this multi-institutional, real-world case series, single-agent ICI demonstrated objective responses and was well tolerated in a heterogeneous treatment-naïve mRCC cohort. ICI monotherapy is not the standard of care for patients with mRCC. With the emergence of several active treatment options for patients with treatment-naïve mRCC, further investigation is necessary to explore predictive biomarkers for optimal treatment selection in this setting.

## Data Availability Statement

The raw data supporting the conclusion of this article will be made available by the authors, if requested.

## Ethics Statement

The studies involving human participants were reviewed and approved by Tulane University Institutional Review Board. Written informed consent for participation was not required for this study in accordance with the national legislation and the institutional requirements.

## Author Contributions

PB: concept. WH, PB, VK, AD, HE, AB, DK, and MB: data collection. All authors: data analysis and interpretation.

## Conflict of Interest

PB has a consulting/advisory role (Institution) with Exelixis, Caris, Bayer, Janssen, EMD/Serono, Pfizer, Astellas, Dendreon, Clovis, and Sanofi. Contracted Research (Institution) from Seattle Genetics, BlueEarth Diagnostics, Nektar, and AstraZeneca. AD serves as an advisory board member for Dendreon and is also on the NCCN Kidney panel. VK has a consulting/advisory role with Janssen, AstraZeneca, Dendreon, Gerson Lehrman Group, Guidepoint Global, and Seattle Genetics/Astellas. Contracted Research (Institution) from Clovis Oncology, Nektar, and Endocyte. OS has a consulting/advisory role with AAA, Astellas, AstraZeneca, Bayer, Blue Earth Diagnostics, EMD Serono, Endocyte, Pfizer, Progenics, Sanofi, Invitae, Merck, Novartis, Janseen, Constellation, Dendreon, BMS, Bravarin Nordic, Clovis, Myriad, Noria Therapeutics, Noxopharm, Point Biopharma, and Tenebio. Contracted Research from Innocrin, Sotio. He also serves as a consultant on NCI Scientific Board Counselors and is a co-chairman of GU Committee at NRG. AB has a consulting/advisory role with EMD Serono and Dendreon. DK has served as an advisory board member for Exelixis, Pfizer, Sanofi, and Merck and has received institutional grants from Exelixis, Astellas. MB has acted as a paid consultant for and/or as a member of the advisory boards of Exelixis, Bayer, BMS, Eisai, Pfizer, AstraZeneca, Janssen, Genomic Health, Nektar, and Sanofi and has received grants to his institution from Xencor, Bayer, Bristol-Myers Squibb, Genentech/Roche, Seattle Genetics, Incyte, Nektar, AstraZeneca, Tricon Pharmaceuticals, Peleton Therapeutics, and Pfizer for work performed as outside of the current study. The remaining authors declare that the research was conducted in the absence of any commercial or financial relationships that could be construed as a potential conflict of interest. The handling editor declared a past co-authorship with several of the authors PB, VK, and AB.

## Abbreviations

AE: adverse events
CTLA-4: cytotoxic T-lymphocyte-associated protein 4
CR: complete response
ICI: immune checkpoint inhibitor
mRCC: metastatic RCC
PD: progressive disease
PD-1: programmed death 1 receptor
PD-L1: programmed death ligand 1
PFS: progression-free survival
PR: partial response
ORR: objective best response rate
OS: overall survival
RCC: renal cell carcinoma
SD: stable disease
TKI: tyrosine kinase inhibitor.
